# The Gut–Brain–Muscle Axis: Microbial Regulation of Neuromuscular Aging and Cognitive Frailty

**DOI:** 10.3390/microorganisms14061366

**Published:** 2026-06-19

**Authors:** Nurpudji Astuti Taslim, Jeremy Nicolas Sibarani, Ricky Indra Alfaray, Nelly Mayulu, Arifa Mustika, Dian Aruni Kumalawati, Happy Kurnia Permatasari, Raymond Rubianto Tjandrawinata, Fahrul Nurkolis

**Affiliations:** 1 Division of Clinical Nutrition, Department of Nutrition, Faculty of Medicine, Hasanuddin University, Makassar 90245, Indonesia; 2 Faculty of Medicine, Universitas Airlangga, Surabaya 60131, Indonesiafahrul.nurkolis.mail@gmail.com (F.N.); 3 Medical Research Center of Indonesia, Surabaya 60281, Indonesia; 4 *Helicobacter pylori* and Microbiota Study Group, Institute of Tropical Disease, Universitas Airlangga, Surabaya 60131, Indonesia; 5 Department of Nutrition, Faculty of Health Science, Muhammadiyah Manado University, Manado 95249, Indonesia; 6 Department of Anatomy, Histology, and Pharmacology, Faculty of Medicine, Universitas Airlangga, Surabaya 60131, Indonesia; 7 Department of Biomedical Sciences, Faculty of Sciences and Technology, State Islamic University of Sunan Kalijaga (UIN Sunan Kalijaga), Yogyakarta 55281, Indonesia; 8 Department of Biochemistry and Biomolecular, Faculty of Medicine, Brawijaya University, Malang 65145, Indonesia; 9 Center for Pharmaceutical and Nutraceutical Research and Policy, Atma Jaya Catholic University of Indonesia, Jakarta 12930, Indonesia; 10 Institute for Research and Community Service, State Islamic University of Sunan Kalijaga (UIN Sunan Kalijaga), Yogyakarta 55281, Indonesia

**Keywords:** gut–brain–muscle axis, cognitive frailty, sarcopenia, gut microbiota, neuromuscular aging, microbial metabolites, neuroinflammation, mitochondria, myokines, precision nutrition

## Abstract

Cognitive frailty, characterized by the coexistence of physical frailty and cognitive impairment, has emerged as a major challenge in aging populations and is closely linked to sarcopenia, neurodegeneration, and chronic inflammation. Increasing evidence suggests that the gut microbiota acts as a central regulator of neuromuscular and neurocognitive aging through the integrated gut–brain–muscle axis. This review highlights how microbial dysbiosis, reduced short-chain fatty acid (SCFA) production, systemic endotoxemia, and altered microbial metabolites contribute to mitochondrial dysfunction, neuroinflammation, anabolic resistance, and impaired neuroplasticity. Key signaling mediators, including SCFAs, bile acids, tryptophan-derived metabolites, cytokines, and myokines such as irisin, brain-derived neurotrophic factor (BDNF), and cathepsin B, orchestrate bidirectional communication among the gut, skeletal muscle, and brain. We further discuss the role of exercise-induced microbiota remodeling and muscle endocrine signaling in promoting mitochondrial biogenesis and cognitive resilience. In addition, emerging translational strategies including probiotics, prebiotics, postbiotics, polyphenol-rich functional foods, marine bioactives, and precision nutrition are explored as potential interventions targeting this axis. Collectively, the gut–brain–muscle axis provides a novel systems biology framework for understanding cognitive frailty and developing integrated therapeutic strategies for healthy longevity.

## 1. Introduction

Aging should be viewed not just as isolated organ decline but as a network-wide process. Brain health, skeletal muscle integrity, and gut physiology are deeply interconnected [1,2,3]. For example, a recent review introduces the notion of “gut frailty”, in which gastrointestinal dysfunction and microbial dysbiosis are posited as central drivers of frailty through the gut–brain and gut–muscle axes [4]. This systems view is supported by evidence that gut microbes influence metabolic, neural, and immune pathways across the body [4,5].

Cognitive frailty (CF) is defined as the co-occurrence of physical frailty and cognitive impairment (often CDR = 0.5) in non-demented older adults [6]. Individuals with CF exhibit weaker muscle strength (sarcopenia or dynapenia), slower gait, and mild cognitive deficits that are not explained by Alzheimer’s disease or other dementia [6,7,8]. This condition carries a high burden; CF predicts greater risk of disability, hospitalization, and progression to dementia. Epidemiologically, CF affects up to ~5–10% of community elders (higher in institutionalized settings) and substantially increases healthcare costs. CF is closely associated with sarcopenia and subtle neurodegeneration; for instance, frail elders often have concurrent declines in muscle mass and executive function, suggesting shared pathophysiology [4,9,10].

Why is the gut microbiota central in this context? The gut–brain axis is a well-recognized bidirectional communication system between enteric microbes and the CNS via neural (vagus nerve/ENS), endocrine, immune, and metabolic pathways [5,11,12]. Adding skeletal muscle creates a triad; skeletal muscle acts as an endocrine organ (secreting myokines and metabolic fuels) that influences both the gut and the brain, and in turn is shaped by gut-derived metabolites and neural signals. Recent reviews explicitly propose a gut–muscle–brain axis, noting that gut dysbiosis has been linked to Alzheimer’s and Parkinson’s pathogenesis and that muscle and microbiota influence each other (the “gut–muscle axis”) [13,14,15]. Physical activity exemplifies this concept, as exercise simultaneously alters gut microbiota composition and muscle secretory profiles, converging on cognitive outcomes [5,13]. Thus, this expanded axis highlights the gut microbiota as a hub connecting neuromuscular health.

This review synthesizes evidence on microbial regulation of neuromuscular aging and cognitive frailty via the gut–brain–muscle axis. We examine how gut microbes affect muscle protein synthesis, mitochondrial function and neuroinflammation; how myokines and metabolites modulate brain aging; and how these pathways intersect. This study focuses on emerging molecular mediators (SCFAs, tryptophan metabolites, myokines, inflammatory cytokines, mitochondrial signals) and discusses translational approaches from functional foods and pro/prebiotics to exercise-microbiome synergy and precision nutrition. The goal of this review is to highlight the integrative systems biology of cognitive frailty and outline future directions for targeting the gut–brain–muscle network in healthy longevity.

## 2. Conceptual Framework of the Gut–Brain–Muscle Axis

### 2.1. Biological Architecture of the Axis

**Figure 1:**
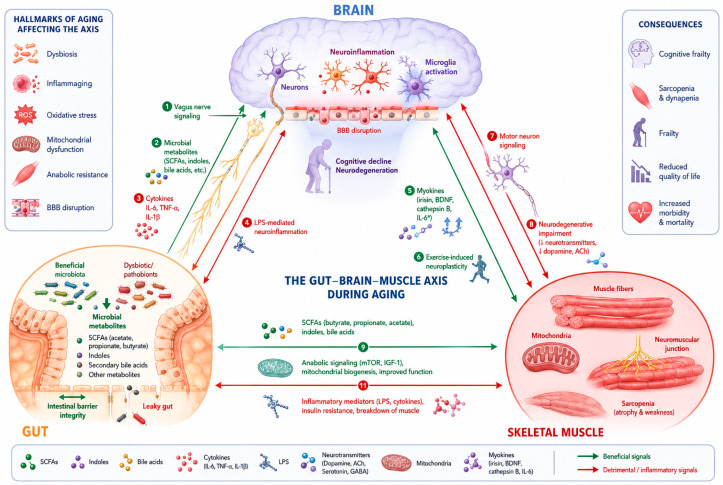
Conceptual overview of the gut–brain–muscle axis, a schematic showing bidirectional signals (vagus, cytokines, metabolites, myokines) among gut, muscle, and brain during aging.

The gut–brain–muscle axis consists of three interconnected bidirectional pathways (Figure 1). Gut ⇄ Brain: The classic gut–brain axis involves neural (vagus nerve, spinal afferents), endocrine and immune routes. For example, gut microbes produce neurotransmitters and metabolites that signal via the vagus nerve or by entering the circulation [5,16,17]. Gut ⇄ Muscle: Gut bacteria modulate muscle through metabolites, SCFAs and bile acids enter the bloodstream to influence muscle metabolism, while muscle activity (exercise) alters gut ecology (favoring SCFA-producing bacteria) [5]. Brain ⇄ Muscle: Skeletal muscle releases myokines (e.g., irisin, cathepsin B, IL-6) that affect brain function (neurogenesis, neuroplasticity) [5]. Conversely, central signals (e.g., via the autonomic nervous system) influence muscle tone and endocrine output, creating feedback loops.

### 2.2. Bidirectional Communication Systems

Four major communication channels operate along these axes: neural pathways, e.g., the vagus nerve transmits gut microbial signals to brainstem nuclei, and muscle afferents communicate to the CNS; endocrine pathways, where the gut and muscle secrete hormones and peptides (gut hormones, myokines such as irisin, BDNF, cathepsin B, IL-6) that act on distant tissues [5]; immune pathways, where cytokines (IL-6, TNF-α, IL-1β, IL-10) and microbial products (LPS) enter the circulation and shape neuroimmune and muscle immune responses [4,5]; and metabolic pathways, where microbial metabolites (SCFAs, secondary bile acids, indole derivatives, ketone bodies, lactate) and muscle-derived fuels (lactate, ketones) serve as biochemical signals. For instance, exercise raises blood lactate and β-hydroxybutyrate (ketones) that fuel neurons, while gut-derived SCFAs bind GPCRs or enter mitochondria to influence host energy balance [5,18,19].

### 2.3. Hallmarks of Axis Dysfunction During Aging

Aging disrupts each component of the axis (Figure 1). Gut dysbiosis: Elderly microbiomes show reduced diversity and loss of key commensals (*Bifidobacterium*, *Faecalibacterium*) with expansion of pathobionts (Enterobacteriaceae) [5,20]. Chronic inflammation (inflammaging): Microbe-driven inflammation via LPS-TLR4 and inflammasomes becomes systemic. Blood–brain barrier (BBB) disruption: Circulating inflammatory mediators and microbial toxins (LPS, p-cresol) compromise the BBB, priming microglia [4,21,22]. Muscle anabolic resistance: Aging blunts the IGF-1/mTOR signaling in muscle; dysbiosis exacerbates this by raising inflammation. Neurodegenerative signaling: Age shifts tryptophan metabolism toward neurotoxic kynurenine, increasing oxidative stress and the senescence-associated secretory phenotype (SASP), which are common to sarcopenia and cognitive decline [4,5]. Together, these alterations create a feed-forward cycle: an impaired gut barrier and mitochondria enhance systemic inflammation, which in turn damages the muscle and the brain (Figure 1).

## 3. Gut Microbiota in Neuromuscular Aging

### 3.1. Aging-Associated Gut Dysbiosis

With advancing age, gut microbial communities remodel substantially. Studies show reduced alpha diversity and a loss of beneficial SCFA-producers (e.g., *Faecalibacterium prausnitzii*, *Roseburia*), along with the relative enrichment of pro-inflammatory taxa (*Escherichia/Shigella*, *Enterobacteriaceae*, *Fusobacterium*) [5,20]. For instance, sarcopenic subjects exhibit a 10–20% lower Firmicutes/Bacteroidetes ratio and a 25–40% depletion of SCFA-generators [5]. Meta-analyses indicate frail elders have higher counts of various pathogens and lower counts of commensals compared to non-frail controls, even if overall diversity metrics vary [20,23,24]. These compositional shifts undermine gut metabolic output (less butyrate and propionate) and barrier integrity.

### 3.2. Microbiota and Skeletal Muscle Homeostasis

**Figure 2:**
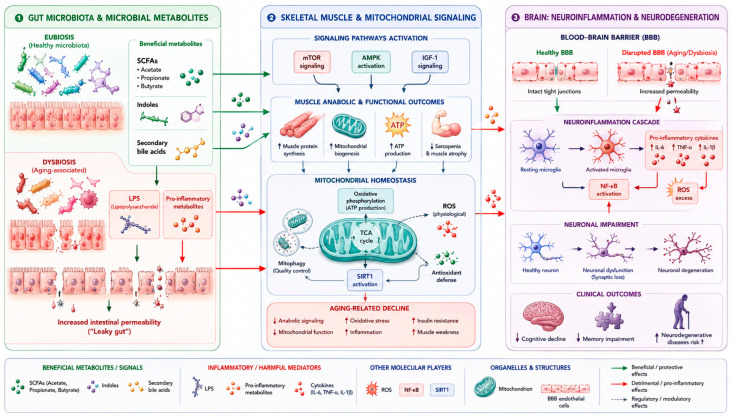
Microbial metabolites regulating muscle, mitochondria, and brain inflammation. Pathways illustrate SCFAs, indoles, and bile acids affecting muscle protein synthesis and neuroinflammation.

Gut microbes influence key anabolic pathways in muscle. SCFAs like butyrate serve as both energy substrates and epigenetic regulators (Figure 2); butyrate enters muscle cells, inhibits histone deacetylases (HDACs) and stimulates protein synthesis. In rodents, butyrate promotes mitochondrial biogenesis (via PGC-1α) and muscle repair. Conversely, dysbiotic increases in Gram-negative bacteria elevate circulating LPS, which binds TLR4 on muscle to activate NF-κB, MuRF1 and Atrogin-1 (ubiquitin ligases), driving atrophy [5,25,26]. The gut microbiota also affects muscle via endocrine cross-talk, for example, microbial production of amino acids and vitamins can influence IGF-1 signaling. Importantly, experiments show causal links: fecal transplants from young donors into aged or sarcopenic mice improve muscle mass and strength. In humans, preliminary trials of FMT in sarcopenic elders raised the abundance of *Faecalibacterium* and *Roseburia*, reduced inflammation (IL-6, TNF-α) and LPS levels, and increased muscle mass and grip strength, suggesting translational promise [5,27,28,29].

### 3.3. Microbial Metabolites in Muscle Aging

Key microbial metabolites mediating gut–muscle interactions include short-chain fatty acids (SCFAs), secondary bile acids, tryptophan/indole derivatives, and endotoxins. SCFAs (acetate, propionate, and butyrate) arise from fiber fermentation. In muscle, butyrate in particular enhances insulin sensitivity and protein synthesis. SCFAs also support muscle mitochondria; they serve as fuel and upregulate PGC-1α (via SIRT1 activation) to promote biogenesis [5,26,30,31]. The aging-related decline in butyrate production contributes to mitochondrial dysfunction and oxidative stress in muscle [4]. Secondary bile acids produced by gut flora (e.g., isoallo-lithocholic acid) modulate muscle metabolism indirectly through FXR/TGR5 receptors and by shaping immune tone; centenarian microbiota uniquely generate secondary BAs that confer anti-inflammatory benefits. Indoles and tryptophan metabolites: Gut conversion of tryptophan yields indole-3-propionic acid and indoxyl-sulfate, which have opposing effects: some indoles activate AhR to dampen inflammation, whereas others (e.g., indoxyl sulfate and p-cresol) exert mitochondrial toxicity [4,32,33]. Lipopolysaccharide (LPS): Systemic LPS from Gram-negative overgrowth binds TLR4 on muscle, triggering catabolic signaling (NF-κB and ubiquitin ligases) [5,34]. Collectively, these metabolites form a chemical “language” by which the microbiome tunes muscle homeostasis (Table 1).

### 3.4. Gut Dysbiosis and Sarcopenia

Evidence linking dysbiosis to sarcopenia is growing. Animal studies show that aged microbiota transplanted into young mice induces muscle weakness, whereas supplementing SCFAs or probiotics preserves muscle (e.g., acetate/propionate supplementation mitigated muscle loss in aging rats) [5,35,36]. Human cohort studies find correlations between microbial signatures and muscle metrics, for example, elderly women with sarcopenia had significantly lower serum propionate and isovalerate. Metabolomic profiling in frail elders often reveals depleted SCFAs and elevated uremic toxins. In a 2025 case–control study, sarcopenic subjects showed reduced propionic and isovaleric acids, highlighting gut-derived metabolites as potential biomarkers [37,38,39]. Overall, both observational and interventional data support a model in which gut dysbiosis through altered metabolite flux and inflammation contributes to age-related muscle decline.

## 4. Gut Microbiota and Cognitive Frailty

### 4.1. Neuroinflammation and Microglial Activation

Neuroinflammation is a key feature of cognitive aging, and the gut microbiota strongly regulates brain immune homeostasis. Systemic LPS and pro-inflammatory cytokines from the gut can reach the brain, activating microglia. For example, translocated LPS accumulates in cerebral vessels and exacerbates neural injury [4,40,41]. In aging, microglia become “primed” by chronic peripheral inflammation, leading to exaggerated responses. Conversely, SCFAs from a healthy microbiota attenuate neuroinflammation; acetate and butyrate cross the BBB or signal via the vagus, thereby suppressing microglial activation and promoting an anti-inflammatory phenotype. It has been shown that SCFAs inhibit microglial pro-inflammatory signaling and reduce IL-6/TNF-α production [5,42,43]. Additionally, the beneficial gut flora stimulates the release of anti-inflammatory cytokines (e.g., gut-derived IL-10) that can indirectly protect the brain. Dysbiosis, by contrast, leads to higher circulating TNF-α, IL-6 and IL-1β, which are associated with cognitive decline and Alzheimer’s pathology. In frail elders, ileal biopsies show increased IL-6 (and decreased TNF/IL-1β) compared to young adults, reflecting dysregulated gut immunity [4,44]. Overall, chronic low-grade neuroinflammation driven by gut-derived signals is a hallmark of cognitive frailty.

### 4.2. Microbial Regulation of Neurotransmitters

Gut microbes also influence brain neurotransmitter systems. Certain bacteria produce or modulate GABA, serotonin, dopamine and glutamate precursors. For instance, *Lactobacillus* and *Bifidobacterium* species synthesize GABA in the gut lumen; animal studies show that oral administration of such probiotics alters GABA_A receptor expression in the hippocampus and amygdala, affecting anxiety and memory via the vagus nerve [45,46,47]. Similarly, gut microbes influence serotonin by shifting tryptophan metabolism; some bacteria increase the conversion of tryptophan to peripheral serotonin rather than the neurotoxic kynurenine pathway [45]. Dysbiosis can perturb these pathways, for example, by reducing serotonergic bacteria (leading to mood and cognitive symptoms) or increasing kynurenine. Glutamate signaling is also modulated indirectly by microbial glutamate production [48,49]. In summary, microbial communities can supply precursors and neuromodulators that traverse gut–brain circuits, so alterations in the microbiome may contribute to the neurotransmitter imbalances observed in cognitive aging.

### 4.3. Gut Dysbiosis and Neurodegeneration

Accumulating studies link gut dysbiosis to neurodegenerative diseases underlying cognitive frailty (e.g., Alzheimer’s, Parkinson’s, mild cognitive impairment). Systematic reviews conclude that gut dysbiosis is strongly associated with cognitive impairment and biomarkers of neurodegeneration [50]. For example, Alzheimer’s patients often exhibit a reduced abundance of *Bifidobacterium* and *Faecalibacterium* and increased *Proteobacteria*. In Parkinson’s disease, α-synuclein pathology correlates with gut microbial changes [51,52]. Mouse experiments show that transplanting microbiota from patients with cognitive decline into rodents accelerates brain amyloidosis and inflammation. These findings suggest that chronic microbial imbalance may facilitate neurodegenerative processes, although causality in humans remains to be proven.

### 4.4. Microbial Metabolites and Brain Energy Metabolism

Finally, microbial metabolites can influence brain energy and mitochondrial function. For instance, ketone bodies (e.g., β-hydroxybutyrate) produced during fasting or ketogenic diets can be modulated by gut flora and are an important neural fuel; however, dysbiosis may impair ketone availability. SCFAs themselves enter neuronal mitochondria and can upregulate oxidative metabolism. Butyrate, apart from its anti-inflammatory action, supports mitochondrial biogenesis via PGC-1α in neurons (similarly to muscle) [5,53,54,55]. The loss of butyrate with age thus exacerbates neuronal oxidative stress [4]. Moreover, microbial metabolites like trimethylamine-N-oxide (TMAO) and advanced glycation end products have been implicated in brain oxidative damage [56,57]. Overall, the gut microbiota influences the brain’s metabolic substrate supply, mitochondrial health, and redox state in ways that can impact cognition.

## 5. Skeletal Muscle as an Endocrine Organ in Brain Aging

### 5.1. Myokines and Brain Function

**Figure 3:**
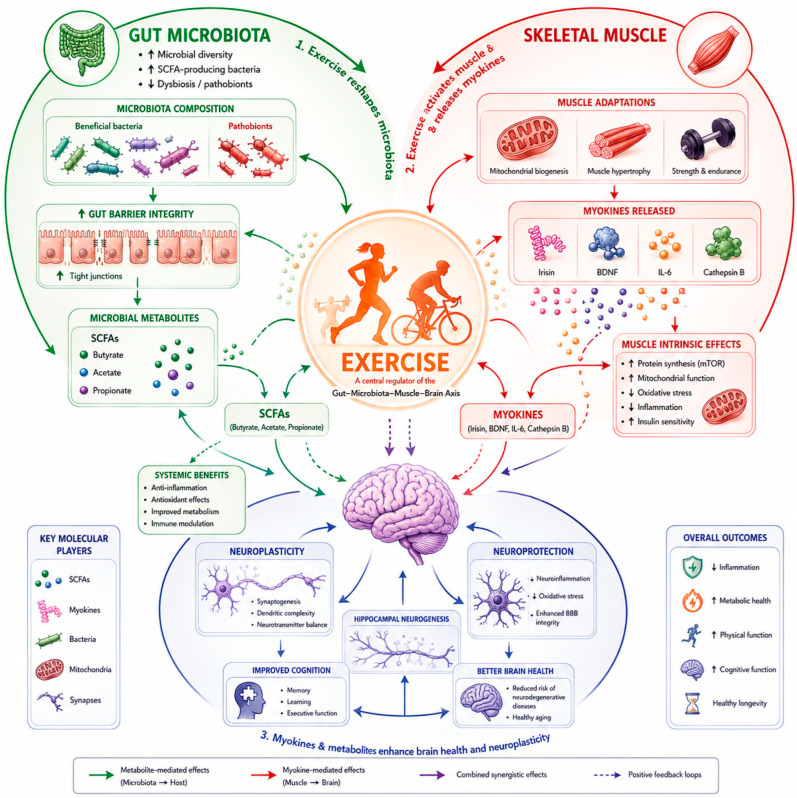
Exercise–microbiota–myokine interaction network.

Skeletal muscle secretes myokines that profoundly affect the brain (Figure 3). Irisin (cleaved from FNDC5 during exercise) crosses the blood–brain barrier and induces hippocampal BDNF expression, enhancing synaptic plasticity and memory [5,58,59]. Brain-derived neurotrophic factor (BDNF) itself is also a myokine induced by exercise, promoting neurogenesis. Cathepsin B, another exercise-induced myokine, is required for running-induced neurogenesis; mice lacking cathepsin B fail to improve memory after exercise. Interleukin-6 (IL-6) has a dual role, acute muscle-derived IL-6 (released during exercise) initiates an anti-inflammatory cascade (IL-6 → IL-10) that confers neuroprotection, whereas chronically elevated IL-6 (from adipose or senescent cells) contributes to neuroinflammation [5,60,61]. Thus, myokines form a muscle–brain endocrine dialog, whereby active muscle sends trophic signals to the brain.

### 5.2. Exercise-Induced Muscle–Brain Crosstalk

Physical exercise powerfully engages the gut–brain–muscle network. Exercise reshapes the microbiota (increasing SCFA-producers and tryptophan-metabolizing species), raising SCFA levels that benefit both the muscle and the brain. The resulting higher SCFAs cross into the CNS, inhibiting microglial activation, upregulating BDNF, enhancing neurogenesis, and modulating neurotransmitters (e.g., serotonin synthesis) [5,62,63,64]. Simultaneously, exercising muscle produces myokines (irisin, cathepsin B) and metabolic fuels (lactate, ketones) that support neuronal metabolism and plasticity. For example, muscle PGC-1α converts excess tryptophan to kynurenine metabolites that do not cross into the brain, thereby preventing stress-induced neurotoxicity. This coordinated response dubbed the neuro-immuno-metabolic (NIM) axis enhances cognitive resilience; exercise triggers energy signals (lactate, ketones) and immune shifts (IL-6 → IL-10) that converge to protect the brain [5,65,66].

### 5.3. Neuromuscular Degeneration and Cognitive Decline

Muscle and brain aging share pathogenic mechanisms. Chronic inflammation (inflammaging) from the gut and adipose tissues promotes both sarcopenia and neurodegeneration [4,5]. Mitochondrial dysfunction in muscle (reduced biogenesis, increased ROS) parallels neuronal mitochondrial decline, impairing function in both tissues [4,5,67]. The “frailty phenotype” often includes both weakness and cognitive slowness, reflecting this shared etiology. For instance, TNF-α and IL-6 not only induce muscle catabolism but also prime microglia and disrupt synapses. Conversely, muscle atrophy deprives the body of beneficial myokines, potentially exacerbating cognitive aging. Thus, neuromuscular and cognitive aging are intertwined manifestations of systemic aging, with muscle–brain crosstalk amplifying frailty.

## 6. Mitochondrial Dysfunction as a Central Integrator

### 6.1. Mitochondria in Aging Brain and Muscle

Mitochondrial decay is a hallmark of aging in both the brain and the muscle. Neurons and myocytes exhibit reduced mitochondrial biogenesis (PGC-1α downregulation) and impaired mitophagy. This leads to lower ATP output and higher reactive oxygen species (ROS) production. In muscle, accumulated mtDNA damage and fission/fusion imbalance contribute to weakness [5,68,69]. Similarly in the brain, mitochondrial dysfunction underlies cognitive impairment and synaptic loss. Studies link sarcopenia to defective oxidative phosphorylation and neuromuscular junction degeneration, mirroring the mitochondrial deficits seen in dementia. Inflammaging and nutrient sensing (e.g., chronic mTOR activation) further exacerbate mitochondrial decline in both tissues.

### 6.2. Microbial Regulation of Host Mitochondria

Gut microbes can modulate host mitochondrial health. SCFAs (notably butyrate) activate SIRT1/PGC-1α pathways, butyrate antagonizes NF-κB and deacetylates PGC-1α to drive mitochondrial biogenesis [5,53]. This enhances oxidative capacity in muscle and may similarly benefit neurons. The microbiota also influence NAD^+^ metabolism, where bacterial production of niacin and other NAD precursors supports SIRT1 activity. For example, niacin supplementation (influenced by gut microbes) has been shown to restore NAD^+^, improving muscle mitochondrial function [5,70,71]. Conversely, dysbiosis reduces SCFAs and NAD^+^, impairing mitochondrial function. Therefore, microbial metabolites act upstream of the SIRT1/PGC-1α axis, directly affecting energy metabolism in the muscle and the brain.

### 6.3. Oxidative Stress and Cellular Senescence

Reduced gut-derived antioxidants and vitamins also heighten oxidative stress. A lack of butyrate and other anti-inflammatory metabolites leads to excess ROS, triggering inflammasomes and the senescence-associated secretory phenotype (SASP) in host cells. Indeed, the age-related butyrate decline results in elevated ROS and disrupted mitochondrial homeostasis [4,72,73]. This oxidative milieu damages proteins, lipids and DNA in the muscle and the neurons, furthering dysfunction. The gut barrier breach (leaky gut) exacerbates the endotoxin burden, compounding ROS production via NADPH oxidases and inflammasomes. Impaired mitophagy in aging (via reduced PINK1/parkin signaling) also allows the accumulation of damaged mitochondria, fueling a vicious cycle of ROS and inflammation [5,74,75]. Together, oxidative stress and senescence are amplified by gut dysbiosis, linking them to both sarcopenia and cognitive frailty.

### 6.4. Mitochondrial Crosstalk in Cognitive Frailty

Ultimately, the shared mitochondrial vulnerability in the muscle and the brain makes it a central integrator of the axis. Toxins and cytokines originating in the gut or muscle affect neuronal mitochondria, while neuronal stress (e.g., β-amyloid) can signal back to peripheral metabolism. For example, systemic LPS can enter the brain and impair neuronal mitochondria, and CNS inflammation can alter the hypothalamic control of muscle metabolism [76,77,78]. In cognitive frailty, interventions that boost systemic mitochondrial health (exercise, SCFA supplements, NAD^+^ precursors) tend to benefit both muscle strength and cognition. This supports the view that maintaining mitochondrial function is key to preserving the gut–brain–muscle axis integrity.

## 7. Immunometabolism and Inflammaging in the Gut–Brain–Muscle Axis

### 7.1. Chronic Low-Grade Inflammation

A hallmark of aging is inflammaging, a persistent rise in circulating pro-inflammatory cytokines. Gut dysbiosis contributes significantly; translocated microbial products (LPS, peptidoglycans) chronically activate immune receptors (e.g., TLR4) in the gut, liver and muscle, sustaining IL-6 and TNF-α release. In muscle, these cytokines drive proteolysis and insulin resistance; in the brain, they activate microglia. Senescent cells (muscle, fat, and endothelium) also secrete SASP factors that overlap with microbial signals [79,80,81]. Thus, gut-originating inflammation and cellular senescence amplify each other across tissues.

### 7.2. Gut Barrier Dysfunction and Leaky Gut

Aging compromises gut epithelial tight junctions, leading to a leaky gut. The reduced production of mucus and butyrate (a key barrier fuel) weakens intestinal integrity. As a result, bacteria and their metabolites (LPS, peptidoglycans, and amyloids) leak into the portal circulation. Elevated serum zonulin (a gut permeability marker) is observed in sarcopenic elders [4,5,82]. This barrier breach is a tipping point: once microbial debris enters the body, it provokes systemic immune activation and the auto-amplification of inflammation [4].

### 7.3. Systemic Endotoxemia

Chronic endotoxemia is a major consequence of a leaky gut. Circulating LPS-binding protein (LBP) and pro-inflammatory cytokines rise with age. LPS binds TLR4 on immune cells, adipocytes, muscle and brain endothelium, maintaining low-grade inflammation. This metabolic endotoxemia is linked to insulin resistance in muscle and the breakdown of the blood–brain barrier. For instance, fecal transplant studies in sarcopenic mice show elevated LPS and NF-κB signaling in muscle [5,79,83]. In the brain, LPS is known to exacerbate amyloid and tau pathology. Detecting LPS, endotoxin-related markers (LBP), or cytokines (IL-6 and CRP) could thus serve as biomarkers of axis dysfunction.

### 7.4. Neuroimmune-Muscle Immune Interactions

Finally, immune signaling between the brain and the muscle adds complexity. During exercise, muscle IL-6 stimulates IL-10 release, which circulates to promote anti-inflammatory microglial phenotypes [2,5,61]. Conversely, brain injury or stress can trigger hypothalamic signals that alter muscle glucose uptake and atrophy signaling. Shared immune mediators (e.g., IL-6 and IL-15) influence both muscle regeneration and microglial activity. Emerging evidence also suggests that gut-derived immune cells may traffic to the muscle or the brain in aging. In sum, the gut–brain–muscle axis is a network of immunometabolic crosstalk; chronic metabolic stress and inflammation in one domain reverberate systemically.

## 8. Multi-Omics Insights into the Gut–Brain–Muscle Axis

Advances in multi-omics are revealing molecular signatures of the gut–brain–muscle axis. Metabolomics analyses identify key metabolites linking tissues, for instance, profiling of blood and muscle in frail elders shows decreased SCFAs and tryptophan derivatives [37,39,84]. Metagenomics (shotgun sequencing) can pinpoint microbial gene pathways altered in sarcopenia and dementia; one study found species-level changes in frail subjects that correlate with inflammatory pathways. Transcriptomics of muscle and brain tissues highlights common age-related gene networks (mitochondrial, inflammatory) that vary with microbiome composition. Proteomics and myokinomics (e.g., muscle secretome analyses) are beginning to map how exercise or probiotics change circulating myokines and cytokines [1,85,86].

Furthermore, network medicine and AI-driven approaches (machine learning, digital twin models) are being applied to integrate multi-omics data. For example, computational modeling of host-microbe metabolic networks can predict how personalized dietary interventions will alter SCFA production and muscle mass. Deep learning on large aging cohorts may identify novel microbe–metabolite–cognition associations. These systems biology tools promise to discover biomarkers (microbial gene sets, circulating metabolite panels) and personalize strategies for gut–brain–muscle health.

## 9. Functional Foods and Therapeutic Modulation of the Gut–Brain–Muscle Axis

### 9.1. Probiotics and Psychobiotics

**Figure 4:**
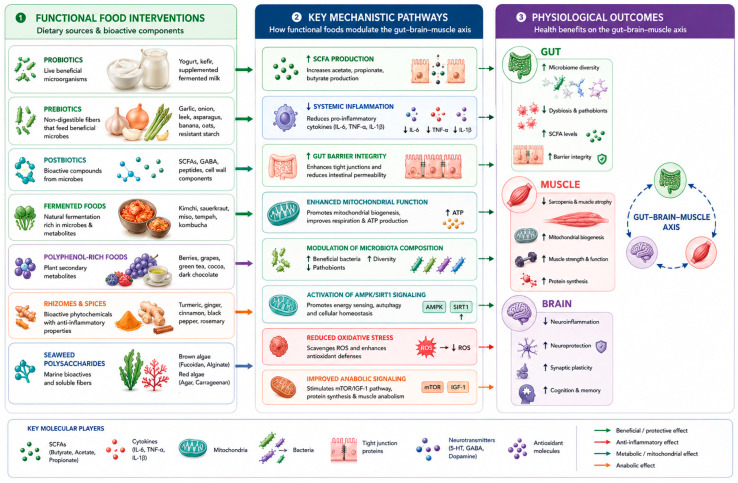
Functional food interventions targeting the gut–brain–muscle axis.

Probiotics (live beneficial bacteria) and psychobiotics (microbes targeting mental health) are prime interventions (Figure 4 and Table 2). *Bifidobacteria* and *Lactobacilli* strains have been shown to increase SCFA levels, strengthen the gut barrier, and modulate myokine release. Clinical trials indicate that probiotic supplementation improves memory and mood in elderly and MCI patients, and can reduce inflammatory markers. Early evidence suggests that probiotics may also slow muscle loss via their anti-inflammatory effects. Psychobiotics (e.g., *Lactobacillus rhamnosus JB-1*) specifically act on the gut–brain axis to elevate neurotrophic factors and reduce anxiety [45,87,88]. Overall, targeted probiotic cocktails hold promise for dual muscle–brain benefits.

### 9.2. Prebiotics and Dietary Fibers

Dietary fibers and prebiotics (inulin, FOS, and galactooligosaccharides) fuel SCFA-producing microbes. Increasing the intake of soluble fiber (from fruits, vegetables, whole grains) boosts butyrate and propionate levels, which, as discussed, support muscle anabolism and reduce neuroinflammation. Novel prebiotics include resistant starches and polyphenol-rich foods (e.g., berries, nuts) that promote *Akkermansia* and *Faecalibacterium*. Early studies in older adults link high-fiber diets to preserved muscle mass and slower cognitive decline, a relationship likely mediated by the microbiota [89,90,91].

### 9.3. Postbiotics

Postbiotics refer to microbial metabolites or inactivated microbes. Supplementing with SCFAs (e.g., sodium butyrate) or microbial extracellular vesicles can bypass the need for live bacteria. For example, butyrate supplements have improved muscle strength and insulin sensitivity in animal models [30,92]. Other postbiotics, like oral bacteriocins or enzymes (e.g., intestinal alkaline phosphatase to degrade LPS), are under investigation to reinforce the barrier function and reduce inflammation [93,94].

### 9.4. Polyphenols and Marine Bioactives

Plant polyphenols (flavonoids, curcumin, resveratrol) and marine polysaccharides act as microbiome modulators. They often function as prebiotics, selectively enhancing beneficial taxa. Notably, seaweed polysaccharides (fucoidan and laminarin) and fermented foods (kimchi and yogurt) enrich SCFA-producing bacteria. Traditional rhizomes (ginger and turmeric) contain compounds (gingerols and curcuminoids) that reduce gut and brain inflammation via microbiota alterations [95,96]. Flavonoids such as quercetin and epigallocatechin gallate have been shown to improve muscle mitochondrial function and cognitive performance, partly through gut microbiota remodeling. Marine bioactives, like astaxanthin and omega-3 fatty acids, also modulate the gut flora and reduce inflammaging [97,98,99].

### 9.5. Exercise-Nutrition-Microbiome Synergy

Combining exercise with dietary modulation yields synergistic benefits. Resistance training improves muscle myokine profiles, while concurrent prebiotic feeding maximizes SCFA production. For instance, protein supplementation combined with probiotic yogurt better preserves muscle in elders than intervention alone. Exercise may also enhance the colonization of beneficial microbes from the diet [100,101]. This tripartite synergy among physical activity, tailored nutrition, and microbiome targeting represents a frontier in geriatric care.

### 9.6. Personalized Nutrition Strategies

Finally, precision nutrition aims to individualize interventions based on an individual’s microbiome and genetics. Gut microbial sequencing can guide personalized diet plans (e.g., specific fibers or polyphenol foods). Machine learning models (digital twins) are being developed to predict who will respond best to a given probiotic or diet. This precision approach promises to optimize the gut–brain–muscle axis for each person, overcoming one-size-fits-all limitations.

## 10. Clinical Translation and Biomarker Opportunities

### 10.1. Biomarkers of Cognitive Frailty

Potential biomarkers span microbes, metabolites and host factors. Microbial signatures: Loss of butyrate-producers (e.g., *Faecalibacterium*) or increase in Enterobacteriaceae in stool could signal risk. Circulating metabolites: Low serum propionate and isovalerate have been associated with sarcopenia [24,37,102]. Similarly, reduced plasma acetate and butyrate may correlate with cognitive impairment. Inflammatory markers: Elevated IL-6, TNF-α, LPS-binding protein, and zonulin indicate axis dysfunction [5,103]. Imaging/functional markers: Brain MRI (showing hippocampal atrophy) and muscle ultrasound (low muscle quality) combined with microbiome/metabolome data might form composite indices of frailty. Developing reliable biomarker panels is an active area, with metabolomics and metagenomics offering high-content signatures.

### 10.2. Precision Medicine Opportunities

The gut–brain–muscle concept opens precision medicine avenues. For example, microbial profiling could stratify individuals for targeted psychobiotics or senobiotics (agents targeting senescent cell-inflammation) to prevent cognitive frailty. Fecal microbiota transplantation might be personalized through donor selection. Drugs modulating specific microbial pathways (e.g., AhR agonists influenced by indoles) could be repurposed for aging [104,105]. Moreover, digital health platforms (wearables measuring strength/cognition) combined with microbiome tracking may enable dynamic, adaptive interventions.

### 10.3. Challenges in Clinical Translation

Translating this science faces hurdles. Heterogeneity: Individual differences in genetics, the microbiome, and lifestyle make it hard to generalize. Causality: Much evidence is correlative, and establishing cause-effect in humans is challenging. Mendelian randomization and longitudinal studies are needed. Microbiome variability: The gut ecosystem is dynamic and influenced by diet, drugs, etc., which complicates interventions. Compliance: Long-term dietary or probiotic regimens show mixed adherence. Clinical trials must carefully control for confounders (e.g., polypharmacy, nutrition). Finally, there is a need for standardized metrics of cognitive frailty and robust biomarkers to assess interventions’ success.

## 11. Future Perspectives

### 11.1. Next-Generation Psychobiotics for Aging

The future will likely see engineered psychobiotics, including designer bacteria or consortia selected to produce specific neurometabolites. Genetically modified probiotics that secrete BDNF or degrade toxic metabolites are being envisioned. In vivo evolution or synthetic ecology approaches could yield strains tailored to the elderly gut environments.

### 11.2. Microbiome-Based Therapeutics

Beyond probiotics, there are prospects for microbiome drugs, such as small molecules that modulate microbial enzymes (e.g., TMA-lyase inhibitors), phage therapies targeting harmful bacteria, or prebiotic compounds with precise microbial targets. Preclinical models (zebrafish, organoids) will accelerate the screening of these interventions for muscle and cognitive endpoints.

### 11.3. AI-Driven Predictive Aging Models

Artificial intelligence will help integrate large multi-omics datasets into predictive models of aging. For instance, digital “twin” simulations of a patient’s gut–muscle–brain system could forecast disease onset or tailor therapy. Machine learning may uncover novel axes (e.g., microbiota–circadian interactions) relevant to frailty.

### 11.4. The Future of Precision Longevity Medicine

Ultimately, the gut–brain–muscle axis perspective aligns with the vision of precision longevity, incorporating interdisciplinary strategies (nutritional genomics, metabolomics, exercise physiology, and data science) to keep people not just alive, but cognitively sharp and physically strong into extreme age. By treating cognitive frailty as a systems biology phenomenon, clinicians and researchers can craft holistic, personalized programs to sustain healthy aging.

## 12. Conclusions

Aging-related cognitive frailty arises from complex inter-organ crosstalk. The gut microbiota emerges as a master regulator of neuromuscular and cognitive health through its metabolites, including SCFAs, indoles, and bile acids. It influences muscle anabolism, mitochondrial function and neuroinflammation. Skeletal muscle is no mere bystander but an endocrine organ, secreting myokines (irisin, BDNF, IL-6, cathepsin B) that modulate brain plasticity. In turn, brain signals shape muscle and gut physiology. Together this forms a gut–brain–muscle network in which inflammaging and mitochondrial decline unite to drive frailty.

Targeting this network via diet, probiotics, exercise, and precision medicine offers new strategies for prevention and therapy. Cognitive frailty should be viewed as a systems-level outcome of gut–muscle–brain interactions. Future research must break organ silos and embrace multidisciplinary approaches (microbiome research, metabolomics, gerontology, and neuroscience) to fully harness the gut–brain–muscle axis for healthy longevity.

## Figures and Tables

**Table 1:** Key microbial metabolites involved in neuromuscular aging.

Microbial Metabolite	Main Source	Major Target	Mechanistic Effects	Relevance to Aging/Frailty
SCFAs: acetate, propionate, butyrate	Fiber fermentation by beneficial gut bacteria	Muscle, mitochondria, brain	Activate AMPK/SIRT1/PGC-1α, support mitochondrial biogenesis, enhance insulin sensitivity, reduce inflammation	Decline of SCFA-producing bacteria may promote sarcopenia, mitochondrial dysfunction, and cognitive decline
Butyrate	Fermentation of resistant starch and dietary fiber	Gut barrier, muscle, brain	Maintains epithelial integrity, inhibits HDACs, suppresses NF-κB, supports protein synthesis and neuroprotection	Reduced butyrate contributes to leaky gut, inflammaging, and impaired muscle–brain resilience
Secondary bile acids	Microbial bile acid metabolism	Muscle metabolism, immune system, brain	Modulate FXR/TGR5 signaling, metabolic homeostasis, and inflammatory tone	Dysregulated bile acid profiles may impair metabolic flexibility and promote inflammation
Indoles and tryptophan metabolites	Microbial tryptophan metabolism	Brain, immune system, mitochondria	Activate AhR, regulate neuroimmune balance, influence serotonin/kynurenine pathways	Balanced indoles may be protective, while toxic metabolites such as indoxyl sulfate may worsen mitochondrial stress
LPS	Gram-negative pathobionts	Muscle, BBB, microglia	Activates TLR4/NF-κB, increases IL-6/TNF-α/IL-1β, induces muscle catabolism and neuroinflammation	Central mediator of endotoxemia, anabolic resistance, BBB disruption, and cognitive frailty
p-cresol and related uremic toxins	Microbial amino acid fermentation	BBB, brain, mitochondria	Promote oxidative stress, vascular dysfunction, and neuroinflammatory signaling	May contribute to cognitive impairment and systemic frailty
TMAO	Microbial metabolism of choline/carnitine	Vascular-brain axis, inflammation	Promotes oxidative stress, endothelial dysfunction, and inflammatory signaling	Potential contributor to neurovascular aging and cognitive decline

**Table 2:** Functional foods and nutraceuticals targeting the gut–brain–muscle axis.

Intervention Category	Examples	Main Bioactive Components	Mechanistic Targets	Potential Outcomes
Probiotics	*Lactobacillus*, *Bifidobacterium*, probiotic yogurt/kefir	Live beneficial microbes	Increase SCFAs, improve gut barrier, reduce cytokines, modulate neurotransmitters	Reduced inflammation, improved cognition, possible muscle preservation
Psychobiotics	*Lactobacillus rhamnosus*, *Bifidobacterium* strains	Neuroactive microbial strains	GABA signaling, vagus nerve signaling, BDNF modulation	Improved mood, memory, neuroplasticity
Prebiotics	Inulin, FOS, GOS, resistant starch, dietary fiber	Fermentable fibers	Increase butyrate/propionate, enrich beneficial bacteria	Improved gut barrier, reduced neuroinflammation, enhanced muscle metabolism
Postbiotics	Sodium butyrate, microbial peptides, inactivated microbes	SCFAs, bacterial cell components, metabolites	Activate AMPK/SIRT1, reduce NF-κB signaling	Mitochondrial support, anti-inflammatory effects, improved muscle function
Fermented foods	Kimchi, tempeh, kombucha, yogurt, kefir	Organic acids, peptides, polyphenols	Improve microbial diversity and SCFA production	Better gut health, reduced dysbiosis, metabolic resilience
Polyphenol-rich foods	Berries, green tea, cocoa, grapes	Flavonoids, EGCG, anthocyanins, resveratrol	Antioxidant effects, microbiota remodeling, AMPK/SIRT1 activation	Reduced oxidative stress, improved mitochondrial function and cognition
Rhizomes and spices	Turmeric, ginger, cinnamon, black pepper	Curcuminoids, gingerols, cinnamaldehyde	Suppress NF-κB and inflammatory cytokines	Anti-inflammatory and neuroprotective effects
Seaweed polysaccharides	Fucoidan, laminarin, alginate, carrageenan	Marine polysaccharides and soluble fibers	Prebiotic-like activity and immune modulation	Improved microbiome diversity and reduced inflammaging
Omega-3 and marine bioactives	Fish oil, algae-derived omega-3, astaxanthin	EPA, DHA, carotenoids	Anti-inflammatory signaling and mitochondrial protection	Reduced neuroinflammation and improved resilience
Exercise-nutrition combinations	Resistance training plus protein/probiotic/prebiotic foods	Amino acids, microbes, fibers	Stimulate myokines, SCFAs, mTOR/IGF-1	Improved muscle strength, cognition, and healthy aging

## Data Availability

No new data were generated in this study. All information discussed is available within the article and in the published papers cited in the references.
